# The necessity of CT/CTA scans on pediatric cerebrovascular injury after blunt trauma – a single center study

**DOI:** 10.1186/s12873-026-01688-4

**Published:** 2026-07-15

**Authors:** Lukas Krüger, Oliver Kamp, Maximilian Wolf, Katharina Alfen, Jens Theysohn, Marcel Dudda, Lars Becker

**Affiliations:** 1 Department of Trauma Surgery, Hand and Reconstructive Surgery, University Hospital Essen, 45147 Essen, Germany; 2 Department of Orthopedics and Trauma Surgery, Evangelisches Klinikum Gelsenkirchen, 45879 Gelsenkirchen, Germany; 3 Department of Pediatrics I, Neonatology, Pediatric Intensive Care Medicine and Pediatric Neurology, University Hospital Essen, 45147 Essen, Germany; 4 Department for Diagnostic and Interventional Radiology and Nuclear Medicine, University Hospital Bergmannsheil Bochum, 44789 Bochum, Germany; 5 Department of Orthopedics and Trauma Surgery, BG-Klinikum Duisburg, 47249 Duisburg, Germany

**Keywords:** Blunt cerebrovascular injury, Pediatrics, TraumaRegister DGU^®^, CT, Radiation risks, Carcinogenesis

## Abstract

**Background:**

Blunt cerebrovascular injury (BCVI) is a rare but potentially devastating complication of pediatric blunt trauma. Delayed or missed diagnosis may result in ischemic stroke, permanent neurological deficits, or death. To avoid missed injuries, many trauma centers apply liberal computed tomography angiography (CTA) screening strategies, although this may expose children to substantial and potentially unnecessary radiation. Several adult- and pediatric-derived screening tools have been proposed, but their applicability in pediatric trauma populations remains controversial. This study aimed to evaluate the incidence of BCVI, imaging utilization, and the diagnostic performance of pediatric BCVI screening approaches in a German Level I trauma center cohort.

**Methods:**

This retrospective observational cohort study included pediatric trauma patients aged 0–15 years who underwent CT imaging following blunt trauma between January 2005 and December 2020 at a Level I trauma center in Germany. Patients were identified through systematic review of electronic medical records and radiological databases. BCVI was defined as traumatic injury to the carotid or vertebral arteries detected by CTA or magnetic resonance imaging (MRI). Demographic, clinical, imaging, and outcome data were collected retrospectively. The McGovern score was calculated for patients with sufficient available data and evaluated using sensitivity, specificity, positive predictive value (PPV), negative predictive value (NPV), and receiver operating characteristic (ROC) analysis. Additionally, the theoretical applicability of PECARN and Scandinavian pediatric head trauma guidelines was explored to assess potential reductions in CT utilization.

**Results:**

A total of 956 pediatric trauma patients underwent CT imaging, of whom 668 (70%) additionally received CTA. BCVI was diagnosed in 6 patients, corresponding to an incidence of 0.6%. Five injuries were identified by CTA and one by MRI. All BCVI patients presented with focal neurological deficits at initial evaluation. The McGovern score could be calculated in 896 patients. Using a cutoff value of ≥ 3 points, all BCVI cases were correctly identified, resulting in a sensitivity of 100%, specificity of 83.8%, PPV of 4.0%, and NPV of 100%. ROC analysis demonstrated excellent discriminative ability with an area under the curve of 0.960 (95% CI 0.923–0.989). However, the small number of BCVI cases limited statistical power. Theoretical application of PECARN and Scandinavian guidelines suggested that a substantial proportion of CT examinations may have been avoidable.

**Conclusion:**

BCVI is an uncommon but clinically significant injury in pediatric blunt trauma. Despite its low incidence, CTA utilization was high in our cohort, raising concerns regarding potential overuse of imaging and radiation exposure. Pediatric-specific screening tools such as the McGovern score may help identify low-risk patients while maintaining high sensitivity. However, larger prospective multicenter studies are required to validate pediatric BCVI screening strategies and optimize the balance between early diagnosis and radiation avoidance.

## Background

Blunt cerebrovascular injuries (BCVI) in pediatric trauma patients are exceedingly rare. BCVI comprises traumatic injuries of the carotid or vertebral arteries, including intimal tears, dissections, pseudoaneurysms, thrombosis, vessel occlusion, and complete transection. Although uncommon in children, these injuries are clinically highly relevant because missed or delayed diagnosis may result in ischemic stroke, permanent neurological deficits, or death. The clinical presentation is often subtle or delayed, making early identification particularly challenging in pediatric trauma care. Latest literature research has shown an overall incidence of 0.03% to 0.5% [1]. A recent large study of the DGU^®^ Traumaregister has shown an incidence of 0.6% [2]. The delayed diagnosis of BCVI is is a major concern, mainly due to the late onset of symptoms, which increases the risk of stroke and long-term complications. Early recognition and prompt treatment are crucial for improving prognosis and minimizing long-term damage. Due to the potentially devastating consequences of missed BCVI and the lack of reliable early clinical symptoms, many trauma centers apply liberal imaging strategies in children with blunt trauma. However, despite increasing use of vascular imaging, only a small proportion of pediatric trauma patients are ultimately diagnosed with BCVI. This creates a major clinical challenge between avoiding missed vascular injuries and minimizing unnecessary radiation exposure in children. Computed tomography angiography (CTA) is currently considered the “gold standard” for detecting BCVI [3, 4]. Up to 16% of pediatric patients with blunt trauma underwent imaging procedures to identify BCVI, with the majority, around 64% to 71% undergoing CTA [3–5]. Several clinical decision rules and screening frameworks have been proposed to improve patient selection for vascular imaging. In adult trauma care, screening recommendations such as the Eastern Association for the Surgery of Trauma (EAST) guidelines and the Denver or Memphis criteria advocate liberal CTA screening in patients with defined injury patterns or neurological abnormalities to avoid missed BCVI. However, the application of these predominantly adult-derived algorithms to pediatric trauma populations remains controversial, as they may contribute to substantial over-imaging and radiation exposure in children with a very low overall incidence of BCVI. In pediatric trauma care, established head trauma algorithms such as the Pediatric Emergency Care Applied Research Network (PECARN) criteria and the Scandinavian Neurotrauma Committee guidelines are commonly used to guide CT imaging decisions after head injury. However, these tools were not specifically developed for BCVI detection and may therefore inadequately identify children at risk for cerebrovascular injury. In contrast to adult trauma care, validated pediatric-specific BCVI screening tools remain limited.

In recent years, several pediatric-specific screening approaches for BCVI have been proposed. The Utah score represented one of the first pediatric-adapted screening tools but demonstrated limited sensitivity in subsequent validation studies [4]. To improve detection rates, the McGovern score incorporated mechanism of injury as an additional predictor and showed sensitivities above 80% with high negative predictive value in multicenter validation cohorts [6]. More recently, the prospective multicenter ATOMAC study compared multiple adult- and pediatric-derived screening tools in children and demonstrated that the Memphis criteria achieved the highest sensitivity (91.7%), whereas the Utah score showed the highest specificity but missed a substantial proportion of injuries [7]. Based on these findings, the ATOMAC+ consortium subsequently proposed the novel A+ criteria, incorporating specific fracture patterns such as temporal bone, sphenoid, orbital roof, and upper cervical spine injuries as predictors for pediatric BCVI [8]. Several pediatric-specific screening tools have been proposed and validated to varying degrees. However, only the McGovern score could be retrospectively evaluated within our cohort because the variables required for calculation were consistently available in our database. The remaining screening approaches are discussed to provide context regarding the current evolution of pediatric BCVI risk stratification. At the same time, efforts to maximize BCVI detection through increasingly sensitive screening strategies may lead to increased CTA utilization and cumulative radiation exposure in children.

However, concerns have been raised regarding the potential long-term effects of radiation exposure, particularly in pediatric patients. Studies suggest that even minimal radiation exposure may increase the risk of pediatric cancer [9, 10]. Latest studies have shown that non-contrast head computed tomography (CT) already involves substantial radiation exposure. Although CTA may have lower per-scan metrics, it is typically performed in addition to CT, resulting in higher cumulative radiation dose [11]. As there are no guidelines for routine diagnostic workups in pediatric BCVI, there is a need to establish a standardized diagnostic approach because early detection is critical for preventing serious complications, such as ischemic stroke and permanent neurological damage, but is also necessary to provide pediatric trauma patients protection from radiation exposure due to unnecessary CT examinations. The variable presentation and delayed symptom onset in children often lead to missed or delayed diagnoses, increasing the risk of adverse outcomes. Accordingly, a substantial proportion of carotid (76%) and vertebral (67%) artery injuries are only identified upon repeat arteriography performed 7–10 days post-injury [12]. There is a clear need for a pediatric-specific clinical decision tool. Consequently, there remains substantial uncertainty regarding which pediatric trauma patients truly benefit from CTA screening and how unnecessary imaging can safely be reduced. As a first step, this study aimed to re-evaluate established risk factors reported in the current literature and to explore potential additional predictors that may improve understanding of these injuries in children and provide a foundation for the future development of a dedicated clinical tool.

## Methods

### Study design and setting

This retrospective observational cohort study was conducted at a Level I trauma center at a university hospital in Germany. All pediatric trauma patients presenting between January 2005 and December 2020 were screened. The study was approved by the institutional review board (ID 24-11942-BO), and the requirement for informed consent was waived due to the retrospective nature of the study.

### Participants

Eligible patients were identified through a systematic query of the hospital’s electronic medical record system. Inclusion criteria were: Age between 0 and 15 years, history of blunt trauma, undergoing CT of the head and/or neck during initial evaluation. Additionally, patients with an ICD-10 diagnosis code S15.- (“Injury of blood vessels at neck level”) were included to ensure capture of all potential BCVI cases. Patients with incomplete imaging data or without documented radiological reports were excluded from diagnostic performance analyses.

### Definition of blunt cerebrovascular injury

BCVI was defined as any traumatic injury to the carotid or vertebral arteries identified on imaging, including intimal injury, dissection, pseudoaneurysm, occlusion, or transection. Diagnosis was established based on radiological reports from CTA or MRI, interpreted by board-certified radiologists.

Imaging findings were classified according to established criteria for blunt cerebrovascular injury as described in the literature (e.g., McGovern criteria) [4]. CTA was the primary diagnostic modality used for BCVI detection in clinical routine, while MRI findings were included in cases where CTA was inconclusive or not performed.

### Data collection

Patient data were extracted from medical records and entered into a structured database (Microsoft Access^®^). The following variables were collected: Demographics: age, sex; Clinical parameters: Glasgow Coma Scale (GCS), focal neurological deficits; Trauma characteristics: mechanism of injury; Imaging data: type of imaging (CT, CTA, MRI), findings; Clinical course: ICU admission, outcome.

Data were collected across four clinical phases: (A) emergency room and initial management, (B) intensive care unit (ICU), (C) general ward, (D) discharge.

### McGovern score and diagnostic assessment

The McGovern score was calculated retrospectively for all patients with sufficient available data, based on published criteria (896 of 956 patients). Consistent with the original McGovern score publication, a score ≥ 3 was defined as a positive screening result indicating high risk for BCVI and the need for vascular imaging [4]. The diagnostic performance of the McGovern score for identifying BCVI was evaluated using: Sensitivity, Specificity, Positive predictive value (PPV), Negative predictive value (NPV). A 2 × 2 contingency table (true positives, false positives, true negatives, false negatives) was constructed. Associations between McGovern Score categories (score ≥ 3 vs. < 3) and the presence of BCVI were additionally evaluated using Fisher’s exact test due to the small number of BCVI cases. Additional contemporary screening tools, including the recently proposed A+ criteria, could not be retrospectively calculated in our cohort because several required imaging variables, including detailed fracture morphology and ligamentous cervical spine injury patterns, were not consistently documented in the database during the study period.

### Statistical analysis

Statistical analysis was performed using SPSS (Version 28.0, IBM Inc., Armonk, NY, USA).

Continuous variables are presented as means with standard deviations or medians with interquartile ranges, depending on distribution. Categorical variables are presented as counts and percentages.

For diagnostic performance analysis: Sensitivity, specificity, PPV, and NPV were calculated with corresponding 95% confidence intervals. Receiver operating characteristic (ROC) curve analysis was performed to assess the discriminative ability of the McGovern score. The area under the ROC curve (AUC) was calculated with 95% confidence intervals. A p-value < 0.05 was considered statistically significant. Missing data were handled on a case-by-case basis and are reported for each variable.

### Bias and limitations

Potential sources of bias include the retrospective single-center design, possible selection bias due to inclusion of only imaged patients, and incomplete documentation of clinical variables. Additionally, the low number of BCVI cases limits statistical power and generalizability of diagnostic performance estimates.

Further limitation is that this study does not represent all trauma patients but only those undergoing CT imaging. Additionally, the dataset lacks comprehensive details on the morphology, anatomical location, and specific type of carotid injuries, as it merely records their occurrence. Furthermore, recently published pediatric-specific screening models such as the A+ criteria could not be externally validated in our cohort because the retrospective database lacked sufficiently granular radiological information regarding fracture morphology and ligamentous cervical spine injuries. Ideally, comprehensive insights could be gained through prospective randomized studies that utilize a data pool specifically tailored to examining pediatric carotid injuries caused by blunt trauma. These studies should emphasize analyzing injury patterns, diagnostic approaches, and radiation exposure to deepen our knowledge and refine clinical management strategies.

## Results

After screening relevant cases, we identified 956 pediatric patients who underwent immediate CT imaging following blunt trauma and clinical evaluation in the emergency room (ER). Of these, 668 children (70%) received an additional CTA of the carotid and vertebral arteries during the same diagnostic session. Twelve children (1.3%) underwent a contrast-enhanced cranial CT scan, 259 (27.0%) had a non-contrast head CT, and 17 (1.7%) received an isolated spinal CT.

The average age of all patients was 9.7 years. In the BCVI group, the mean age was 10.3 years, compared to 9.0 years in the non-BCVI group. Half of the BCVI cases occurred in children aged 13 to 15 years. No relevant differences in gender distribution were observed between groups: 4 male and 2 female patients in the BCVI group; 577 males and 373 females among non-BCVI patients. The most common trauma mechanism was traffic accidents (44%), followed by falls from height (39.6%). All baseline characteristics of the study population and a summary of the important results are listed in Table 1. A detailed breakdown of trauma mechanisms is provided in Table 2.

**Table 1:** Baseline characteristics of the study population

Variable	Total (*n* = 956)	Non-BCVI (*n* = 950)	BCVI (*n* = 6)
**Age (mean ± SD)**	9.7	9.0	10.3
**Sex**			
Male	581	577	4
Female	375	373	2
**Initial GCS**			
13–15	745	745 (78.4%)	0
9–12	32	31 (3.4%)	1
4–8	12	12 (1.3%)	0
3	108	103 (10.8%)	5
Missing	59	59 (6.1%)	0
**Presenting focal neurological deficit**	154	148	6
**Imaging performed**			
CT + CTA	668	663	5
CT without CTA	288	288	0
**CT findings**			
No pathology	614	614 (64.4%)	0
Hemorrhage	139	139 (14.5%)	0
Skull fracture	233	233 (23.3%)	0
Petrous bone fracture	53	51 (5.3%)	2
**McGovern Score**			
0–2	746	746 (78.5%)	0
3–4	88	87 (9.2%)	1
5–6	62	51 (5.4%)	4
7–8	8	6 (0.6%)	1
Missing	60	60 (6.3%)	0
**Outcome**			
Death			3
Survived			3

**Table 2:** Trauma mechanism

			Non-BCVI	BCVI
Fall of Accident history	Car crash	n	57	-
		%	6%	-
	Car-Pedestrian	n	210	1
		%	22.2%	16.6%
	Car-Bicycle	n	26	1
		%	2.7%	16.6%
	Bicycle	n	71	-
		%	7.5%	-
	Motorcycle	n	38	1
		%	4%	16.6%
	Bus/Truck-Pedestrian	n	8	1
		%	0.8%	16.6%
	Train/Tramway-Pedestrian	n	7	-
		%	0.7%	-
	High fall > 3 m	n	89	1
		%	9.4%	16.6%
	Low fall ≤ 3 m	n	222	-
		%	23.3%	-
	Fall of Horse	n	37	-
		%	3.9%	-
	Other	n	185	1
		%	19.5%	16.6%
Total		n	950	6
		%	100%	100%

**Fig. 1:**
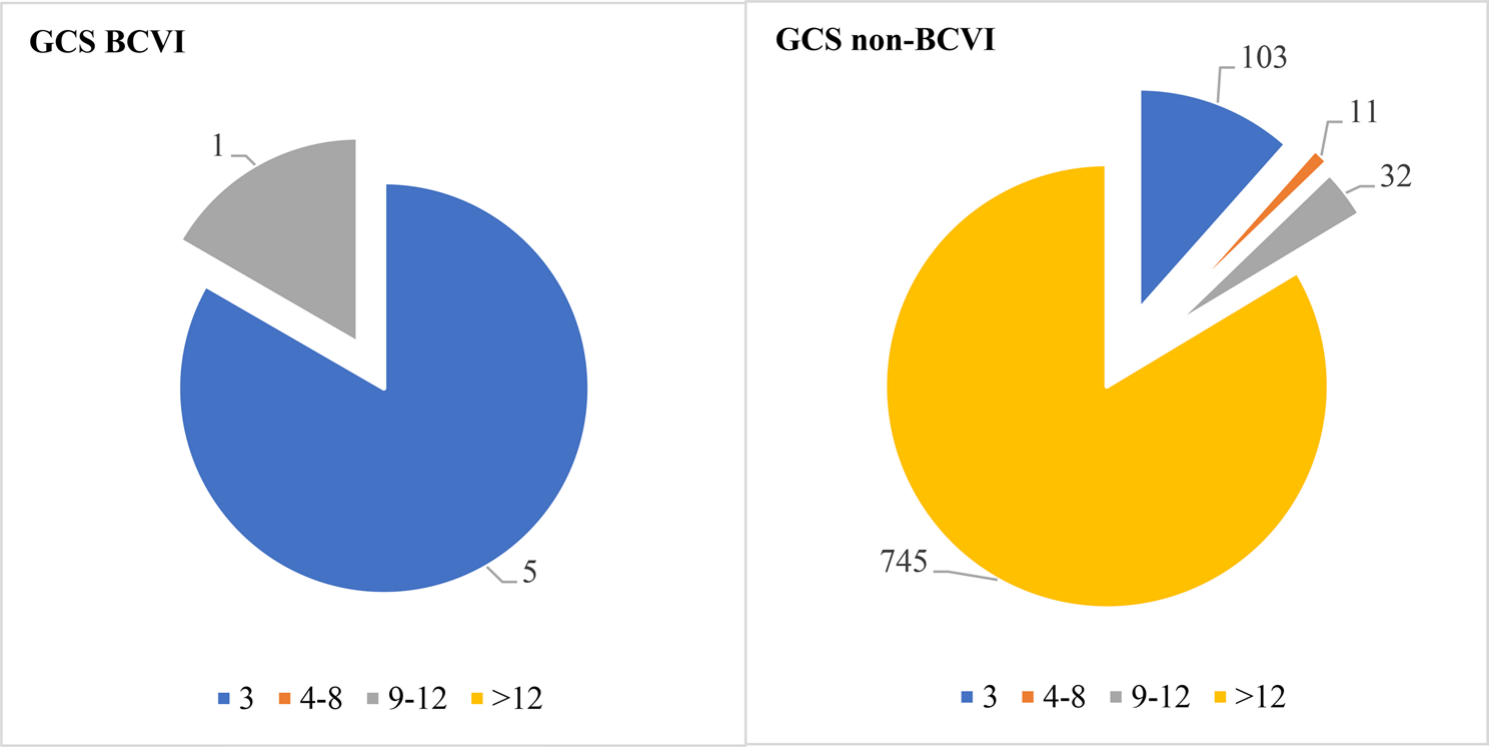
GCS as a possible sreening parameter for BCVI. This figure shows the screening parameter Glasgow Coma Scale (GCS) for two diffrent groups. Compared are trauma patients without BCVI with trauma patients with BCVI. Total *n* = 956, patients without BCVI *n* = 950, patients with BCVI *n* = 6

BCVI was diagnosed in only 6 out of 956 cases (0.6%). Five of these were detected through CTA, and one was identified later by an additional MRI. Detailed clinical characteristics of all six patients with BCVI are summarized in Table 3. Notably, no cases of BCVI were found among patients who underwent CT without CTA, nor among trauma patients who did not receive any radiological imaging. Of the 956 CT scans, 614 (64.4%) revealed no pathological findings. Cerebral hemorrhage—including epidural, subdural, subarachnoid, intracerebral, and contusion bleeding—was identified in 139 scans (14.5%). Skull or midface fractures were diagnosed in 233 cases (23.3%), with calvaria fractures (131 cases, 13.7%) and petrous bone fractures (53 cases, 5.5%) being the most common. In the BCVI group, only two of the six patients had an associated petrous bone fracture. All six BCVI patients presented with a focal neurological deficit on initial ER examination. Among non-BCVI patients, 148 children were diagnosed with such deficits. The initial GCS on arrival in the ER was 3 in five of the six BCVI patients and 12 in one case. In contrast, among non-BCVI trauma patients, 745 had a GCS > 12 (78.4%), 31 had a score between 9 and 12 (3.4%), 12 had a score between 4 and 8 (1.3%), and 103 had a GCS of 3 (10.8%). GCS data were missing in 59 cases (Fig. 1).

**Table 3:** Characteristics of Patients with BCVI (*n* = 6)

Variable	Patient 1	Patient 2	Patient 3	Patient 4	Patient 5	Patient 6
Age (years)	15	16	8	5	13	5
Sex	m	f	m	m	f	m
Mechanism of injury	car/scooter	car/pedest.	car/bicycle	fall>3 m	bus/pedest.	other
Initial GCS	3	3	3	12	3	3
Focal neurological deficit	yes	yes	yes	yes	yes	yes
Diagnostic modality	CTA	CTA	CTA	CTA	CTA	MRI
McGovern score	3	8	6	5	5	6
Petrous bone fracture	no	no	yes	yes	no	no
Associated injuries	-	SAH	SDH, Clavicula #	ICH, SAH	Cerebral eddema	-
Outcome	Alive	Died < 24 h	Alive	Alive	Died < 24 h	Died > 24 h

m = male, f = female, pedest. = pedestrian, CTA = computed tomography angiography, MRI = magnetic resonance imaging, SAH = Subarachnoid hemorrhage, SDH = subdural hematoma, ICH = Intracerebral hemorrhage, # = fracture

**Fig. 2:**
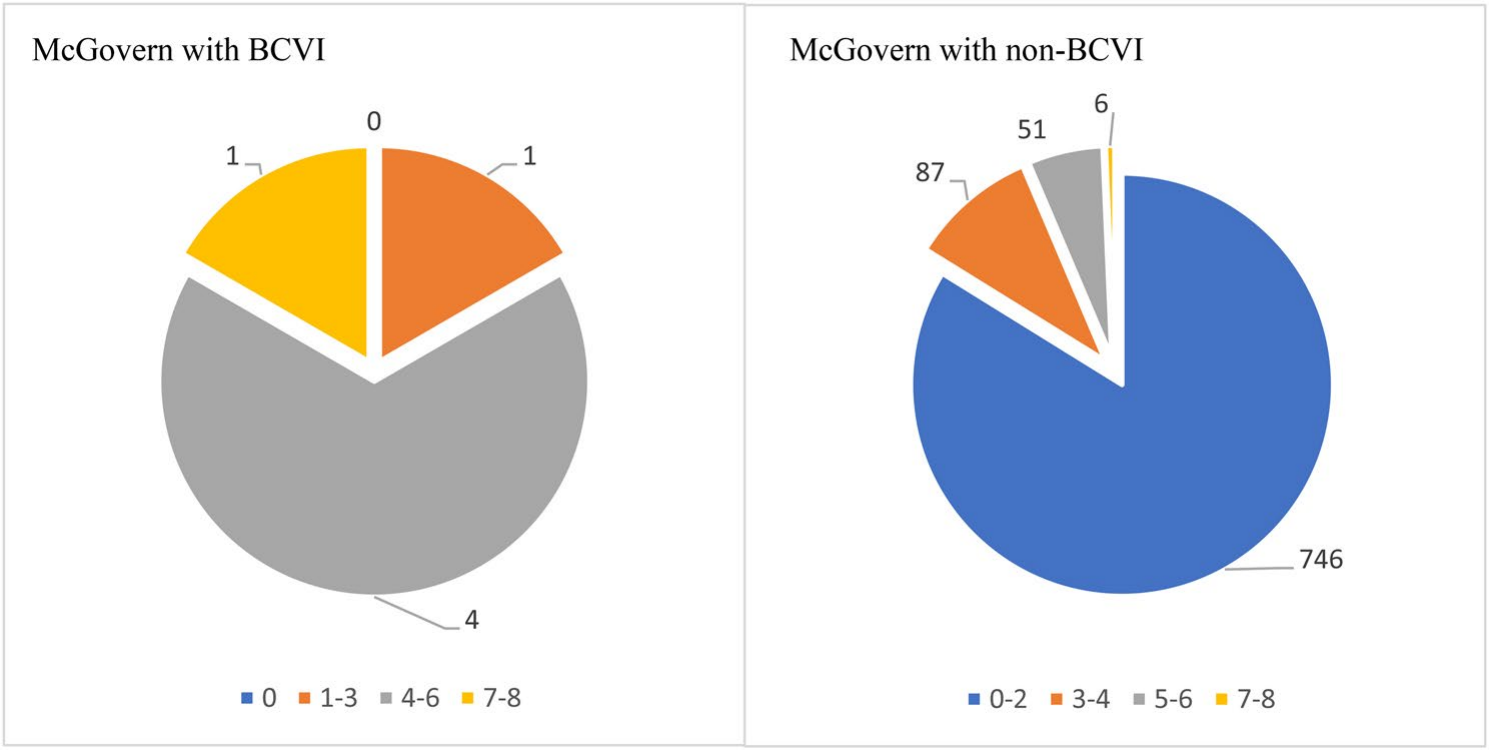
McGovern-score as a possible sreening parameter for BCVI. This figure shows the screening parameter McGovern-score for two different groups. Compared are trauma patients without Blunt cerebrovascular Injuries (BCVI) with trauma patients with BCVI. Total *n* = 956, patients without BCVI *n* = 950, patients with BCVI *n* = 6. In the group of BCVI patients, the McGoverns score was not calculable in 60 cases

The McGovern Score was also assessed. The clinical factors used to calculate the McGovern score are summarized in Table 4. Among patients with confirmed BCVI, one patient had a score of 3 points, two patients scored 5 points, two scored 6 points, and one patient scored 8 points. In the non-BCVI group, 746 patients (78.5%) had scores between 0 and 2 points, 87 patients (9.2%) scored between 3 and 4 points, 51 patients (5.4%) scored between 5 and 6 points, and 6 patients (0.6%) scored between 7 and 8 points. In 60 cases (6.3%), the McGovern Score could not be calculated (Fig. 2).

**Table 4:** McGovern Criteria

GCS score ≤ 8 (1Pt.)
Focal neurological deficit (2Pt.)
Carotid canal fracture (2Pt.)
Petrous temporal bone fracture (3Pt.)
Cerebral infarction on CT (3Pt.)
MOI (2Pt.)
⇒ For McGovern Criteria, a score ≥ 3 points on both scales signifies high risk for BCVI and indicates angiography.

Using the predefined cutoff value of ≥ 3 points, all six BCVI cases were correctly identified, resulting in a sensitivity of 100%. Among non-BCVI patients, 144 were classified as positive and 746 as negative, corresponding to a specificity of 83.8%. The positive predictive value (PPV) was 4.0%, while the negative predictive value (NPV) was 100%. Receiver operating characteristic (ROC) analysis yielded an area under the curve (AUC) of 0.960 (95% CI 0.923–0.989). Given the small number of BCVI cases, this finding should be interpreted as exploratory. A 2 × 2 contingency table summarizing the diagnostic performance of the McGovern Score is presented in Table 5. Fisher’s exact test demonstrated a statistically significant association between a McGovern Score ≥ 3 and the presence of BCVI (*p* < 0.001).

**Table 5:** Diagnostic performance of the McGovern Score for prediction of BCVI

	**BCVI Present**	**BCVI Absent**	**Total**
**McGovern Score ≥3**	6 (TP)	144 (FP)	150
**McGovern Score < 3**	0 (FN)	746 (TN)	746
**Total**	6	890	896 (missing *n* = 60)
**Diagnostic Performance of the McGovern Score**			
**Parameter**	**Value**		
Sensitivity	100%		
Specificity	83.8%		
Positive Predictive Value (PPV)	4.0%		
Negative Predictive Value (NPV)	100%		
AUC (95% CI)	0.960 (95% CI 0.923–0.989)		

TP = true positive, FP = false positive, TN = true negative, FN = false negative, AUC = area under the curve, CI = confidence interval

During hospitalization, 2 of the 6 BCVI patients died within 24 h, one died after 24 h. The remaining three patients were discharged alive; however, no long-term follow-up data were available.

## Discussion

This study aimed to examine the incidence of BCVI in a Level I trauma center in Germany and to evaluate the use of radiological imaging in its diagnosis. We also sought to assess the applicability of existing clinical scores and guidelines in identifying at-risk groups to reduce unnecessary CT scans in pediatric trauma patients.

A review of the literature indicates an overall incidence of BCVI in pediatric blunt trauma ranging from 0.03% to 0.5%, which is consistent with our finding of 0.6% [1, 3, 13–19]. Only two studies have reported higher incidences of 0.9% and 1.1% [20, 21]. Due to its speed, comprehensive imaging capabilities, and high diagnostic accuracy, CT is frequently employed following blunt trauma to detect internal injuries. In fact, 52.5% of all pediatric polytrauma patients undergo CT scanning as part of the initial diagnostic evaluation [22]. Given that CTA is considered the gold standard for BCVI diagnosis, it is not surprising that 70% of the patients who underwent CT also received CTA. Importantly, CTA is typically performed in addition to standard CT imaging rather than as a replacement, thereby substantially increasing cumulative radiation dose in pediatric trauma patients. This issue is further complicated by the widespread adoption of adult-oriented BCVI screening recommendations such as the EAST guidelines, which prioritize high sensitivity to avoid missed vascular injuries. While these approaches may be justified in adult trauma populations with higher BCVI incidence, their transferability to pediatric patients is uncertain. Due to the substantially lower incidence of BCVI in children, strict application of EAST-associated screening criteria may result in a considerable number of unnecessary CTA examinations and increased cumulative radiation exposure.

Recent prospective multicenter pediatric studies have further highlighted the limitations of directly transferring adult screening algorithms to children. In the ATOMAC multicenter cohort, the Memphis criteria demonstrated the highest sensitivity for pediatric BCVI detection (91.7%), outperforming the McGovern and Utah scores, but at the cost of lower specificity and potentially increased CTA utilization [7]. In contrast, the Utah score achieved high specificity but failed to identify more than half of all BCVI cases. These findings underline the persistent challenge of balancing early detection with radiation avoidance in pediatric trauma care.

However, upon reviewing clinical assessments in the emergency room, we found a considerable number of cases in which no clinical risk factors for BCVI were present. This raises concerns about potential overuse of imaging. To further explore this, we examined the applicability of two widely used guidelines for pediatric head trauma: the PECARN (Pediatric Emergency Care Applied Research Network) criteria [23] used on the ATLS guidelines [24] and the Scandinavian guidelines for initial management of minor and moderate head trauma in children [25, 26]. 

**Fig. 3:**
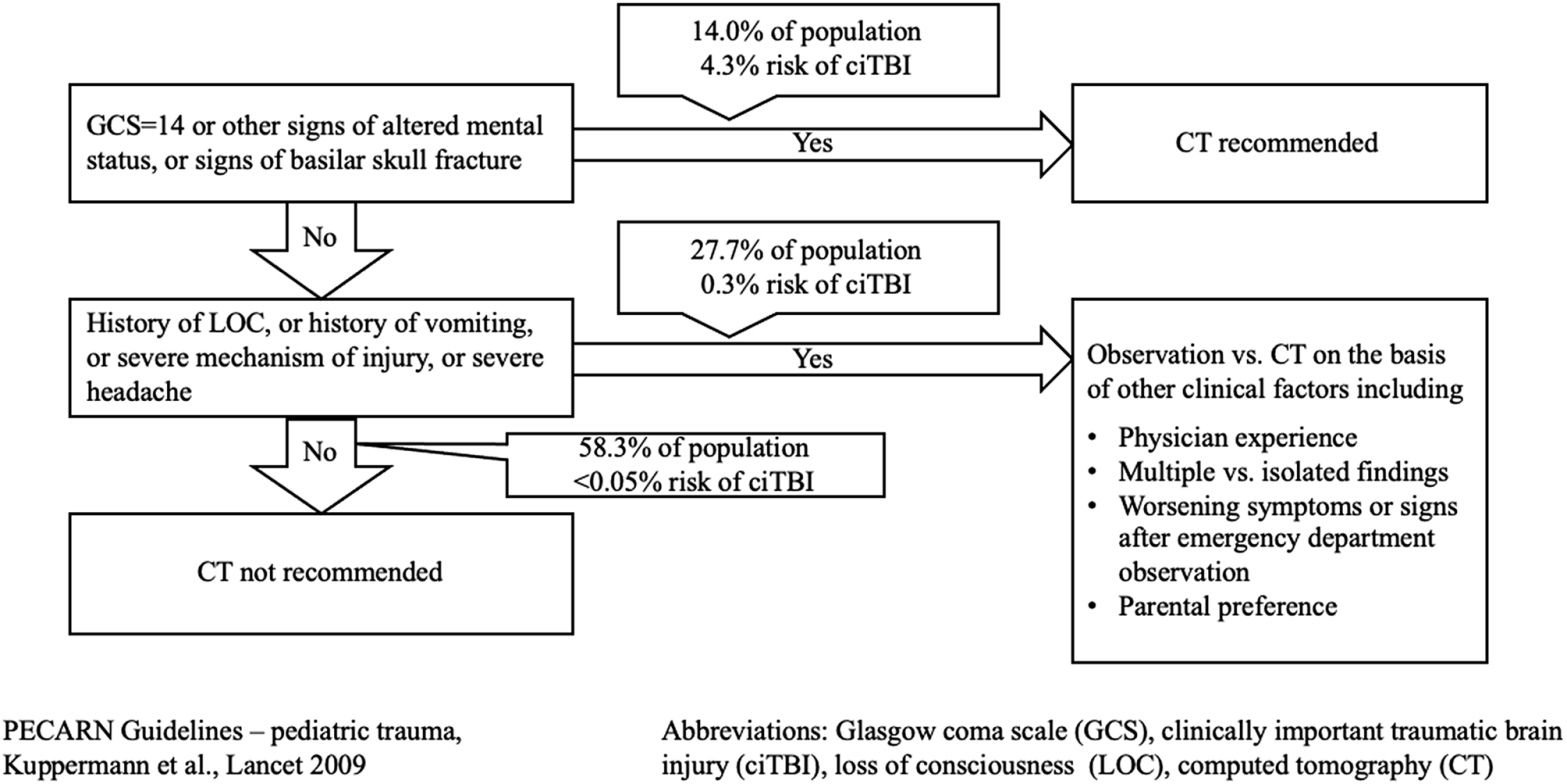
Pediatric Emergency Care Applied Research Network (PECARN) used on ATLS guidlines. Criteria of Head CT[1, 23, 24]

According to PECARN (Fig. 3), following the chart, only patients with a GCS of 14 or other signs of altered mental status, or signs of basilar skull fracture should get a CT scan at first. That should be about 14% of the population. In our dataset, 697 patients (72.9%) with a GCS of 15 underwent CT imaging. Following the chart, the next step would be to look for a history of Loss of consciousness (LOC), history of vomiting, a severe mechanism of injury, or severe headache. Only 8 patients of those with a GCS of 15 had a focal neurological deficit in the ER. The documented trauma mechanism was a high-impact trauma in only 50% of these cases. Following PECARN guidelines, 58% of all pediatric trauma patients should not be recommended to a CT scan. As we do not have a documented number of pediatric trauma cases that did not underwent any CT scan in our database, we cannot confirm this section of the PECARN guidelines.

**Fig. 4:**
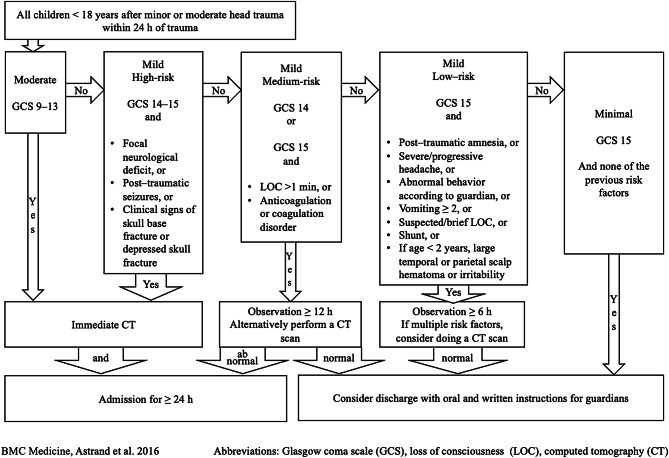
Scandinavian guidelines for initial management of minor and moderate head trauma in children [1, 25]

The Scandinavian guidelines (Fig. 4) recommend immediate CT only for patients with GCS 9–13 or those with GCS 14–15 accompanied by focal neurological deficits, post-traumatic seizures, or clinical signs of skull base or depressed fractures. According to this definition, only 84 of 956 patients matched the criteria for immediate CT. The remainder should have been initially observed. However, because our database lacks exact timestamps for the decision to perform CT scans and the reasoning behind it, we cannot determine how many CT scans were performed after clinical deterioration during observation.

Given the imperative to minimize diagnostic radiation in pediatric populations, it is crucial to critically assess radiation doses from CT, their associated risks, and the availability of alternative imaging modalities. In children, a non-contrast head CT typically delivers a median volume CT dose index of approximately 33 milligray (mGy) [27]. Depending on the anatomical region and age group, CT imaging of the skull or facial bones yields 27–37 mGy, the neck 19–26 mGy, and the petrous bones 42–67 mGy [28]. Notably, CTA involves considerably higher doses, starting at 138 mGy [29]. 

Numerous studies have demonstrated a correlation between ionizing radiation exposure and increased cancer incidence. In 2007, the Center for Radiological Research at Columbia University Medical Center provided epidemiological evidence indicating that organ doses from standard CT scans are linked to elevated cancer risk [9]. Complementary findings from a Dutch cohort study revealed that a cumulative brain dose of 38.5 mGy from pediatric brain CTs was significantly associated with the occurrence of brain tumors [30]. Further support comes from the Institute of Health and Society and the Northern Institute of Cancer Research in Newcastle, which reported a near tripling of leukemia risk in children exposed to cumulative CT doses of approximately 50 mGy. Similarly, brain cancer risk tripled at exposure levels around 60 mGy [31]. Moreover, younger age at the time of exposure and increased lifetime attributable risk reinforce the notion that children are more vulnerable to radiation-induced malignancies than adults [32]. 

Several pediatric-specific screening tools for BCVI have now been proposed, including the Utah score, the McGovern score, and most recently the A+ criteria. The multicenter validation study of the McGovern score demonstrated sensitivities above 80% and negative predictive values exceeding 98%, supporting its utility as a clinically sensitive screening tool [6]. In the past scoring systems, such as the Memphis, Denver, EAST, and Utah-scores, have been found inadequate due to high misclassification rates (ranging from 28.6% to 47.6%) [4, 33–36]. However, subsequent prospective ATOMAC data suggested that the Memphis criteria may provide even higher sensitivity for pediatric BCVI detection, whereas the Utah score showed the highest specificity but substantially lower sensitivity [7]. 

In our dataset, we calculated the McGovern Score for 896 patients. A score ≥ 3 indicates high risk and warrants angiography. All six BCVI cases were correctly identified, with 144 positive and 746 negative results among non-BCVI patients. This yields a sensitivity of 100%, specificity of 83.8%, positive predictive value (PPV) of 4%, and negative predictive value (NPV) of 100%. Similar to previous reports, the observed high negative predictive value may indicate potential usefulness for excluding BCVI in low-risk pediatric trauma patients. These findings should be interpreted cautiously due to the limited number of BCVI events, larger studies are required to confirm this observation. While our findings demonstrate a McGovern Score with a sensitivity of 100%, in contrast, multicenter validation data by Nickoles et al. reported a sensitivity of 75% and specificity of 89.5%, suggesting that real-world performance may be lower in larger cohorts [7]. 

From a practical perspective, retrospective application of the McGovern score cutoff of ≥ 3 would have classified only 150 patients as screening-positive, compared with 668 patients who actually underwent CTA. This suggests that approximately 518 CTA examinations (77.5%) might theoretically have been avoided while still identifying all six BCVI cases in our cohort. Although this estimate must be interpreted cautiously due to the retrospective study design and the small number of outcome events, it highlights the potential value of structured screening approaches for reducing radiation exposure in pediatric trauma patients.

As the McGovern Score includes petrous bone fracture as a risk factor (3 points), we examined its relevance. In our cohort, only 2 of the 6 BCVI cases had a petrous bone fracture, whereas 53 non-BCVI patients did. As a result, this criterion led to a large number of false positives.

Recently, the ATOMAC+ consortium proposed the novel A+ criteria, which incorporate temporal bone fractures, sphenoid fractures, orbital roof fractures, and upper cervical spine injuries as pediatric-specific predictors of BCVI [8]. Unfortunately, retrospective application of these criteria was not possible in our cohort because the necessary detailed fracture morphology and ligamentous injury characteristics were not consistently available in the database.

Finally, ultrasound has been proposed as a promising non-radiative tool for BCVI screening [37–39]. However, in our dataset, no carotid vessel ultrasound examinations were documented; only focused assessment with sonography for trauma (FAST) sonography was recorded during the initial treatment in the ER.

Taken together, current evidence suggests that no single screening tool optimally balances sensitivity and specificity for pediatric BCVI. Adult-derived criteria tend to overestimate risk and increase imaging rates, whereas pediatric-adapted scores such as Utah and McGovern improve specificity but may miss a subset of injuries. Recent multicenter data further indicate that Memphis criteria provide the highest sensitivity, while emerging A+ criteria may represent a next step in refining pediatric-specific risk stratification.

## Conclusion

BCVI is a rare but serious consequence of pediatric blunt trauma, with an incidence of 0.6% in our cohort. Despite this, many children—particularly those with GCS 15 and no neurological deficits—underwent CT/CTA, indicating potential overuse of imaging and unnecessary radiation exposure. All BCVI cases showed focal neurological deficits at initial presentation, supporting their role as a key clinical indicator. Contemporary pediatric BCVI screening tools, particularly the McGovern score, may help reduce unnecessary vascular imaging in selected pediatric trauma patients. In our cohort, retrospective application of a McGovern score cutoff ≥ 3 would theoretically have reduced CTA utilization from 668 examinations to approximately 150 patients, potentially avoiding 518 CTA studies (77.5%) while still identifying all BCVI cases. However, these findings should be interpreted cautiously given the retrospective study design and the very small number of BCVI events. Prospective pediatric-specific validation studies remain necessary to establish reliable screening standards that adequately balance early BCVI detection against radiation exposure. Theoretical application of PECARN and Scandinavian head trauma guidelines suggests additional potential for reducing CT utilization, although these tools were not designed for BCVI detection and require cautious interpretation in this context.

## Data Availability

The data that support the findings of this study are not openly available due to reasons of sensitivity and are available from the corresponding author upon reasonable request.

## Abbreviations

ATLS: Advanced Trauma Life Support
AUC: Area Under the Curve
BCVI: Blunt Cerebrovascular Injury
CI: Confidence Interval
CT: Computed Tomography
CTA: Computed Tomography Angiography
EAST: Eastern Association for the Surgery of Trauma
ER: Emergency Room
FAST: Focused Assessment with Sonography for Trauma
GCS: Glasgow Coma Scale
ICD-10: International Classification of Diseases, Tenth Revision
ICU: Intensive Care Unit
LOC: Loss of Consciousness
MRI: Magnetic Resonance Imaging
NPV: Negative Predictive Value
PECARN: Pediatric Emergency Care Applied Research Network
PPV: Positive Predictive Value
ROC: Receiver Operating Characteristic
SD: Standard deviation
SPSS: Statistical Package for the Social Sciences
